# Assessment of myocardial blood flow, viability and diffuse fibrosis in patients after arterial switch and ross operation with magnetic resonance imaging

**DOI:** 10.1186/1532-429X-17-S1-P103

**Published:** 2015-02-03

**Authors:** Minh H Pham, Philip Wegner, Michael Jerosch-Herold, Inga Voges, Ana C Andrade, Christopher Hart, Ravi V Shah, Hans-Heiner Kramer, Carsten Rickers

**Affiliations:** Pediatric and Adult Congenital Heart Disease, University Hospital of Schleswig-Holstein, Kiel, Germany; Radiology, Brigham & Women’s Hospital, Harvard University, Boston, MA USA; Cardiology, University Medical Center HCMC, Ho Chi Minh, Viet Nam

## Background

Coronary artery reimplantation is crucial step during the arterial switch (ASO) and Ross operation. Mortality and long-term outcome after the operation mainly depend on the patency and function of the reimplanted coronary arteries due to risk of stenosis, stretching, or occlusion.

Cardiovascular magnetic resonance (CMR) imaging has emerged as a promising diagnostic tool for the evaluation of children heart. We utilized advanced CMR methods to perform a noninvasive assessment of myocardial blood flow, viability, function and diffuse fibrosis in patients after ASO and Ross operation to guide further therapy and for a better understanding of the microcirculation.

## Methods

MRI first-pass perfusion imaging (0.03 mmol/kg Gd-DTPA; TR/TE/α=2.6/1.1/20°) was performed in 36 patients (age, 15.75±10.94 yrs; transposition of the great arteries post arterial switch operation n=25, post Ross operation for the treatment of aortic valve disease n= 11) and in 10 age matched healthy controls. Myocardial blood flow (ml/g/min) was calculated in 6 LV segments per slice (2-3 slices/pt). Quantitative blood flow at rest and stress (Adenosin 140 µg/kg/min) was derived from signal intensity curves by model independent deconvolution. Late enhancement studies (Gd 0.1 mmol/kg) using T1 weighted inversion recovery sequences were performed to detect myocardial scar. A Look-Locker technique (temporal resolution, 40 ms; slice thickness, 8 mm; repetition time, 3 R-R intervals) for measurements of T1 was used for detecting of LV diffuse fibrosis. Furthermore, cine MRI and 3 D coronary artery imaging were performed to assess ventricular function and coronary anatomy.

## Results

Of the entire cohort 15 pts (41.7 %) had known or suspected coronary problems such as occlusion, stenosis or hypoplasia. In 7/15 pts (46.7%) we found regional ischemia, scar tissue (28.6%) and regional or global impairment of LV function (30%). As a consequence of our findings one patient received MIDCAB surgery and 4 patients (26.7%) were treated medically.

In pts with patent epicardial coronaries (58.3%) myocardial perfusion reserve (MPR) was significantly reduced as compared to the healthy controls (2.79±0.75 vs. 3.75±1.13; p<0.05) and only in one patient scar tissue was detected. No regional or global wall motion abnormalities were detected. Furthermore, T1 mapping showed increased extracellular matrix expansion suggestive of diffuse fibrosis (0.37±0.09 vs. 0.26±0.01; p<0.02).

## Conclusions

CMR imaging can provide a comprehensive assessment of myocardial perfusion, viability and function in patients after coronary reimplantation to guide further therapy such as surgical revascularisation. Of note, in patients with patent epicardial coronaries, we measured an impaired myocardial perfusion reserve and increased interstitial fibrosis.

## Funding

Kinderherzen-Wollen-Leben e. V, Neumünster, Germany.

